# *N*-glycan Cryptic Antigens as Active Immunological Targets in Prostate Cancer Patients

**DOI:** 10.4172/jpb.1000218

**Published:** 2012-04-30

**Authors:** Denong Wang

**Affiliations:** Tumor Glycomics Laboratory, Center for Cancer Research, Biosciences Division, SRI International, 333 Ravenswood Avenue, Menlo Park, CA 94025, USA

**Keywords:** Autoantibodies, Carbohydrate microarrays, Cryptic antigens, Tumor glycomics, Oligomannose, Prostate cancer

## Abstract

Although tumor-associated abnormal glycosylation has been recognized for decades, information regarding host recognition of the evolving tumor glycome remains elusive. We report here a carbohydrate microarray analysis of a number of tumor-associated carbohydrates for their serum antibody reactivities and potential immunogenicity in humans. These are the precursors, cores and internal sequences of *N*-glycans. They are usually masked by other sugar moieties and belong to a class of glyco-antigens that are normally “cryptic”. However, viral expression of these carbohydrates may trigger host immune responses. For examples, HIV-1 and SARS-CoV display Man9 clusters and tri- or multi-antennary type II (Galβ1→4GlcNAc) chains (Tri/m-II), respectively; viral neutralizing antibodies often target these sugar moieties. We asked, therefore, whether prostate tumor expression of corresponding carbohydrates triggers antibody responses *in vivo*. Using carbohydrate microarrays, we analyzed a panel of human sera, including 17 samples from prostate cancer patients and 12 from men with Benign Prostatic Hyperplasia (BPH). We observed that IgG antibodies targeting the Man9- or Tri-/m-II-autoantigens are readily detectable in the sera of men with BPH, as well as those with cancer. Importantly, these antibody activities were selectively increased in prostate cancer patients. Thus, human immune systems actively recognize these *N*-glycan cryptic carbohydrates and produce targeting antibodies. This finding shads a light on a class of previously less studied immunological targets of human cancers. Identifying the diagnostic, prognostic and therapeutic values of these targets will require further investigation.

## Introduction

Recognition of tumor-associated abnormal glycosylation has raised a great interest in the potential for carbohydrate-based cancer biomarkers. For prostate cancer (PCa), a number of tumor-associated aberrant carbohydrates have been identified and characterized, including the glyco-isoforms of PSA [1–3], sulfated glycolipid [4] and blood group antigens and precursors [5,6]. The latter represent a diverse panel of *O*-glycans. These include, but are not limited to, T antigen [7,8], Tn, sialyl and globo-H [9]; the branched and linear type II backbone regions (I and i antigens); the difucosylated Le^y^ or the monofucosyl, or monosialyl compound of sialyl-Le^x^, blood group H and Le^b^ [10]; type I backbone-based sialyl-Le^a^ [11]; and sialyl-Le^x^ in association with metastatic PCa [12].

While carbohydrate researchers are exploring the diversities of tumor glycome, other investigators have turned their attention to the immunological properties of tumor-associated carbohydrates. Wandall et al. [13] developed an *O*-glycopeptide microarray to monitor human autoantibody responses to tumor antigens that they successfully used to detect cancer-associated IgG autoantibodies against different aberrant *O*-glycopeptide epitopes derived from MUC1 in sera from breast, ovarian and prostate cancer patients. Last year Blixt et al. [13,14] reported that the presence and level of autoantibodies were significantly higher in the sera from cancer patients compared with sera from the control subjects and a highly significant correlation with age was observed. High levels of a subset of autoantibodies to the core3MUC1 and STnMUC1 glycoforms (GlcNAcβ1-3GalNAc-MUC1 and NeuAcα2, 6GalNAc-MUC1, respectively) were significantly associated with reduced incidence and increased time to metastasis. These results suggest that autoantibodies to aberrantly glycosylated MUC1 in early stage breast cancer are associated with a better prognosis.

Padler-Karavani et al. [15] found that human carcinomas can metabolically incorporate the dietary non-human sialic acid Neu5Gc. That monosaccharide differs from the human sialic acid N-acetylneuraminic acid (Neu5Ac) by one oxygen atom but is able to trigger a differential antibody response. Using a novel sialoglycan microarray presenting multiple Neu5Gc-glycans and control Neu5Ac-glycans, these investigators found that antibodies against Neu5Gcalpha2-6GalNAcβ1-O-Ser/Thr (GcSTn) are more prominent in patients with carcinomas than in patients with other diseases. Thus, these xeno-autoantibodies and xeno-autoantigens are considered potential targets for diagnostic, prognostic and therapeutic applications in human carcinomas.

**Figure 1:**
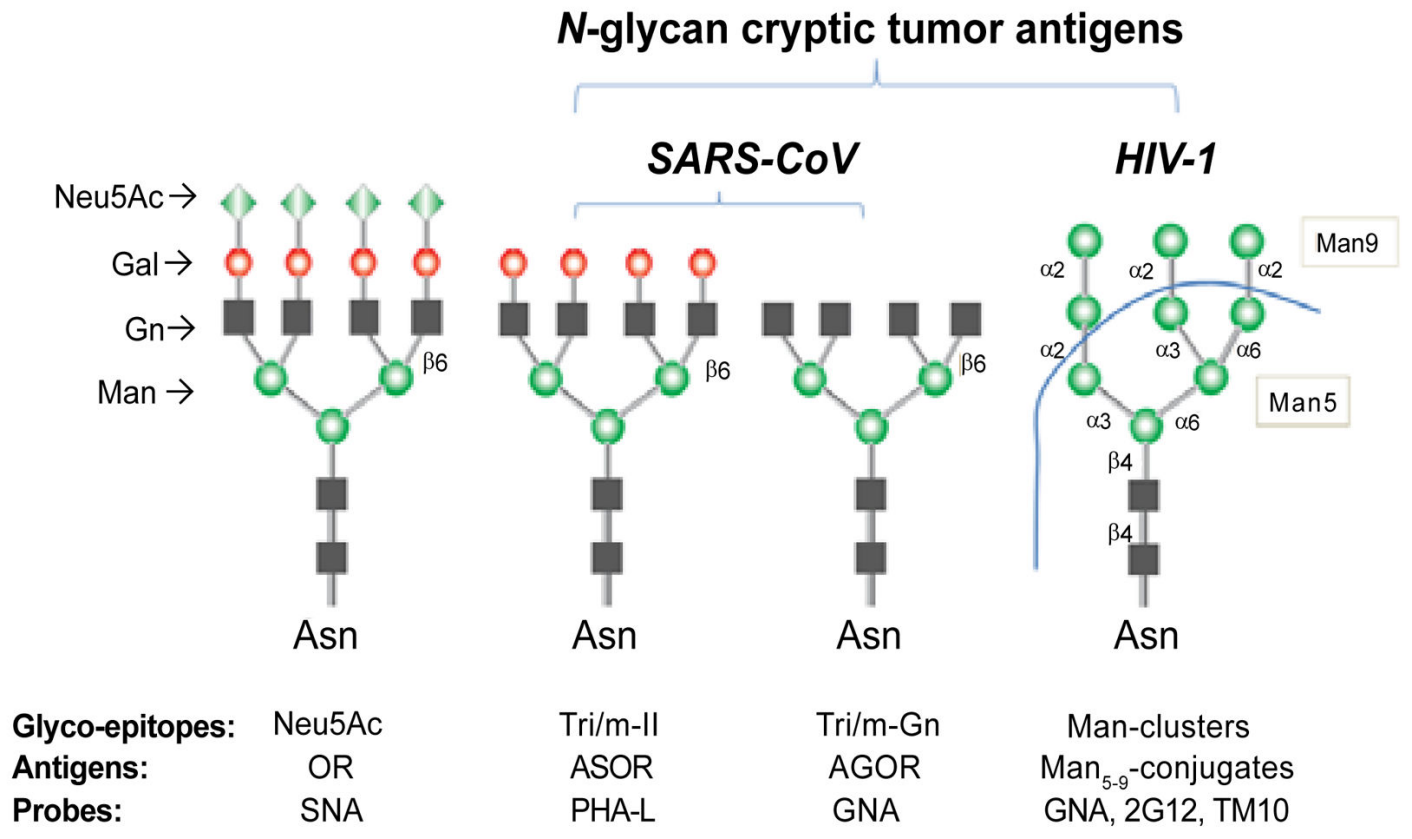
Schematic of a panel of *N*-glycan “cryptic” antigens that are highly expressed by viruses and cancers Expression of Neu5Ac, Tri/m-II, and Tri/m-Gn glyco-epitopes in glycoprotein Orosomucoid (OR), Asialo-OR (ASOR), and Agalacto-OR (AGOR) is readily detectable by lectin *Sambucus nigra* I (SNA), PHA-L, and GNA, respectively. GNA, 2G12, and TM10 recognize different glyco-epitopes of oligomannoses expressed by cancer cells or viruses.

Our laboratory has been investigating a class of cryptic glycan markers that are differentially expressed among different Gleason grades of PCa and metastatic tumors [16–18]. These markers include high-mannose (Man9) clusters; tri-antennary type II or multivalent type II (Tri/m-II) chains; and the agalactosyl derivatives, Tri-/m-Gn (GlcNAc)-glycoepitopes. As illustrated in Figure 1, they share the N-glycan Man-cores but differ in the terminal sugar moieties. Unlike the fully glycosylated cellular *N*-glycans, which are often capped by Neu5Ac, these targets expose the internal sequences that are normally “cryptic” to the immune systems. However, some viral pathogens express these carbohydrates on the surfaces of their virions. For examples, the HIV-1 envelop glycoprotein gp120 are heavily coated with Man9 clusters [19,20] and the spike protein of SARS-CoV expresses Tri/m-II moieties [21]. Importantly, viral neutralizing antibodies often target these carbohydrates [19–23].

One of the open questions is whether host immune systems recognize and respond to the tumor-expressed *N*-glycan cryptic carbohydrates. Newsom-Davis and Wang et al. [24] recently reported that a tumor cell-based vaccine elicited anti-Man9-cluster antibodies. In this experiment, Fas-ligand-transfected melanoma cells were used for animal immunization. Flow cytometry analysis revealed that a monoclonal antibody (mAb), TM10, established by this immunization strategy illustrates a unique binding profile. TM10 does not bind to the cell surface of untransformed normal cells but strongly binds to a number of murine and human tumor cell lines, including those from melanoma, prostate, breast and ovarian cancers. Our carbohydrate microarray analysis revealed that TM10 recognizes the oligomannosyl epitopes presented by two Man9 clusters, (Man9)n-keyhole limpet hemocyanin (KLH) and [(Man9)4]n-KLH [24], which were constructed to investigate the HIV-1 neutralizing epitopes recognized by a human mAb 2G12 [19,20].

These findings promoted us to investigate whether human immune systems recognize and respond to these tumor-associated cryptic glyco-antigens. In the current study, we characterized a panel of sera from men with PCa and sera from men with BPH using carbohydrate microarrays. Our rationale was that if these tumor carbohydrates were immunogenic *in vivo*, cancer patients would be possible to mount antibody responses to corresponding targets. Detection of these antibodies in PCa subjects by carbohydrate microarrays would provide evidence to pin down the specific immunological targets. Results of this study are summarized in this report.

## Materials and Methods

### Serum specimens, antigens and antibodies

A panel of 29 banked human sera specimens for analysis in this study was kindly provided by Dr. Zeqi (Joe) Zhou, of Egenix, Inc. (Millbrook, NY). Diagnosis of PCa (N=17) or BPH (N=12) was based on the results of prostate needle biopsy in a clinic. Surgical Gleason grade information is, however, not available for this cohort. The specimens were blinded before we conducted our analysis.

Cy3-conjugated anti-human IgG used for carbohydrate microarray assay was purchased from Sigma (St. Louis, MO). Carbohydrate antigens, glycoconjugates and other antigen preparations applied in this study are listed in Supplementary Table 1.

### Carbohydrate microarrays

The microarrays used comprised 32 distinct antigen preparations, including 16 potential autoantigens and 16 common environmental antigens. The latter were selected because they often detect significant anti-carbohydrate antibodies in human circulation and can provide control probes for monitoring the global antibody profiles in a given subject. Members of the *N*-glycan cryptic sugar moieties illustrated in Figure 1, including Man-cores, Tri-/m-Gn and Tri/m-II, were presented by a panel of spotted carbohydrate antigens. These include native human glycoproteins OR, ASOR and AGOR, as well as two synthetic high-mannose clusters, (Man9)n-KLH and [(Man9)4]n-KLH [25–28].

For microarray printing, polysaccharides and glycoproteins were dissolved in saline and lipids were prepared as liposomes as described [29–32]. Antigen solutions or liposome suspensions were spotted onto nitrocellulose-coated FAST slides (Schleicher & Schuell) by a high-precision robot designed to produce cDNA microarrays (Cartesian Technologies’ PixSys 5500C). Immediately before use, the printed microarray slides were washed in 1xPBS at room temperature (RT) for 5 min and blocked with 1% BSA-PBS at RT for 30 min. They were incubated with 50 μl of sera (1:25) at RT for 1 hour, washed and then incubated with titrated secondary anti-human IgG antibodies coupled with Cy3 at RT for 30 min. The stained slides were then rinsed five times and air-dried at RT before being scanned. The ScanArray5000A Microarray Scanner (PerkinElmer Life Science) was used to scan the stained microarrays for fluorescent signals. Fluorescent intensity values for each array spot and its background were calculated using ScanArray Express software (PerkinElmer Life Science).

### Microarray data-processing and statistical analysis

Carbohydrate array datasets for 29 microarray assays, including sera from 17 PCa patients and 12 BPH patients, were standardized and statistically analyzed using the JMP Genomics software package from SAS Institute (Cary, North Carolina). The standardized antibody reactivity (IgG) is shown as microarray scores, which are the log2 transformed (median-background) values normalized by setting their interquartile ranges (IQR) to be identical. One-way ANOVA was performed to compare the results obtained among groups and/or subgroups. Student’s t test was applied to calculate the significance of differences among comparative groups.

## Results

**Figure 2:**
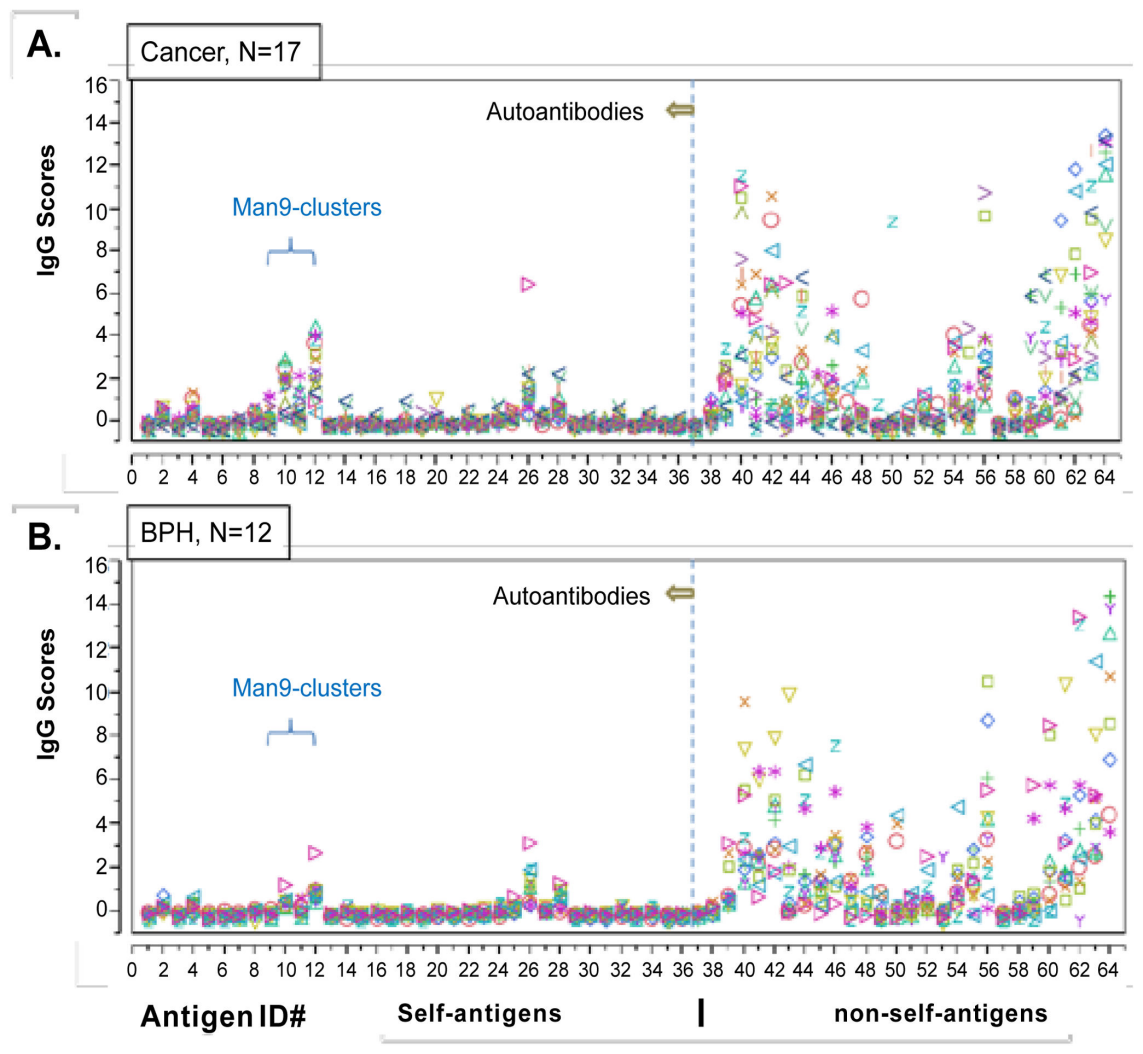
Carbohydrate arrays recognize global similarity in antibody profiles among men with BPH and those with prostate cancer (PCa) Sera from 17 subjects with PCa and 12 with BPH were characterized using carbohydrate arrays. Array datasets were processed and statistically analyzed using SAS Institute’s JMP Genomics software package. Results are presented as overlay plots to illustrate the antibody profiles of each subject and to enable a visual comparison of the two groups, cancer (**A**) vs. BPH (**B**). Each dot in the plot is the mean value (IgG score) of triplicate array detection of a sample. The microarray datasets are presented in Supplemental Table 1.

**Figure 3:**
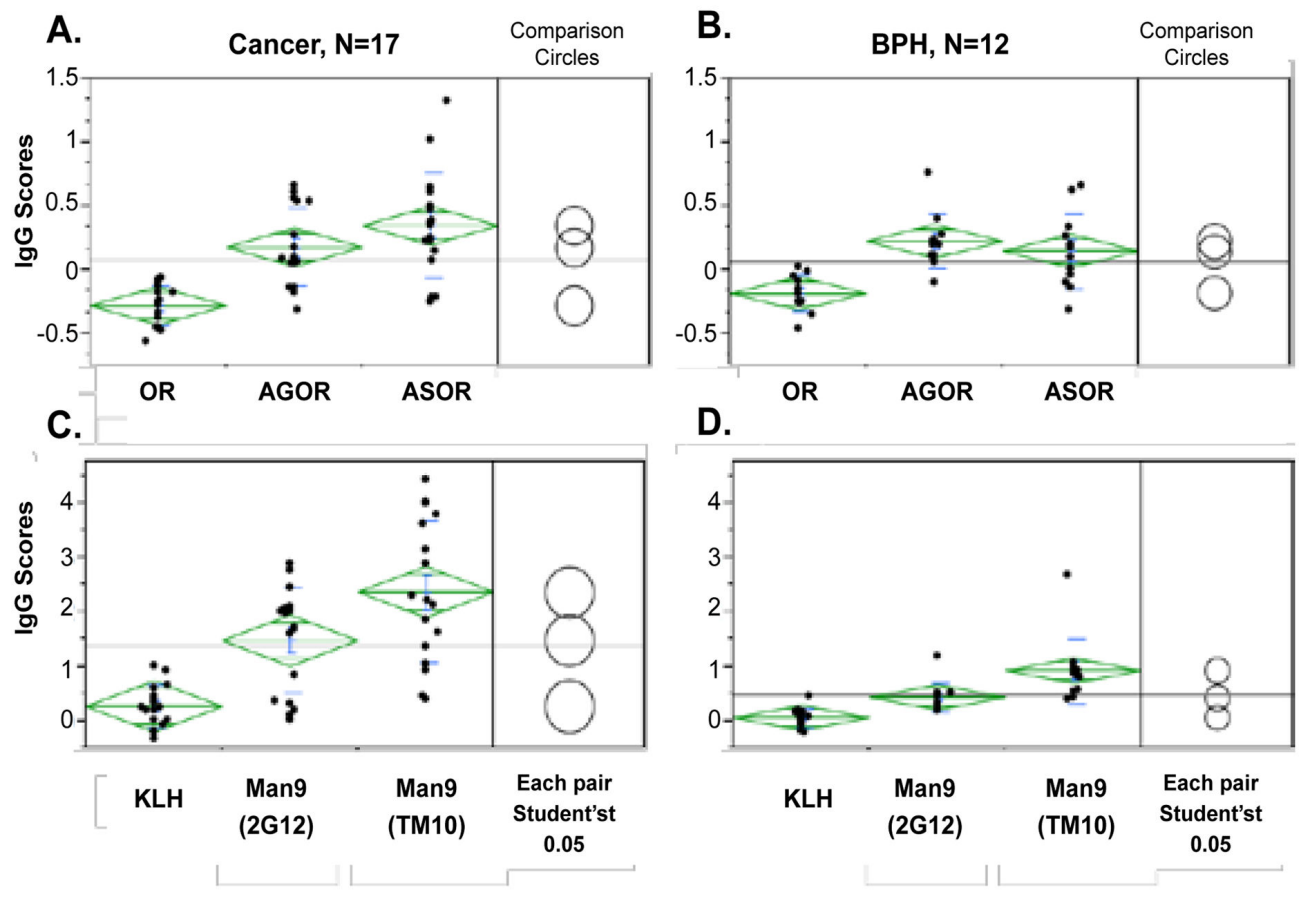
Pair-wise in-group comparisons of the relative IgG activities among selected antigens ***Panel A & B***: OR, AGOR, and ASOR; ***Panel C & D***: KLH, Man9(2G12), and Man9(TM10). ***Panel A & C***: PCa group; ***Panel B & D:*** BPH group. One-way analysis was performed to compare group means among selected autoantigens listed in each panel. Each dot in the panels represents the mean value (IgG score) of triplicate array detection of a subject. The means are shown as bars and standard deviations as green diamonds around the mean value. The comparison circles for Student’s t-tests appear to the right of the means diamonds to illustrate the significance of the differences among the means. By graphically showing the intersections, these circles allow visual inspection of the significance of differences. The more the circles intersect, the less the significance of difference is and vice versa.

**Figure 4:**
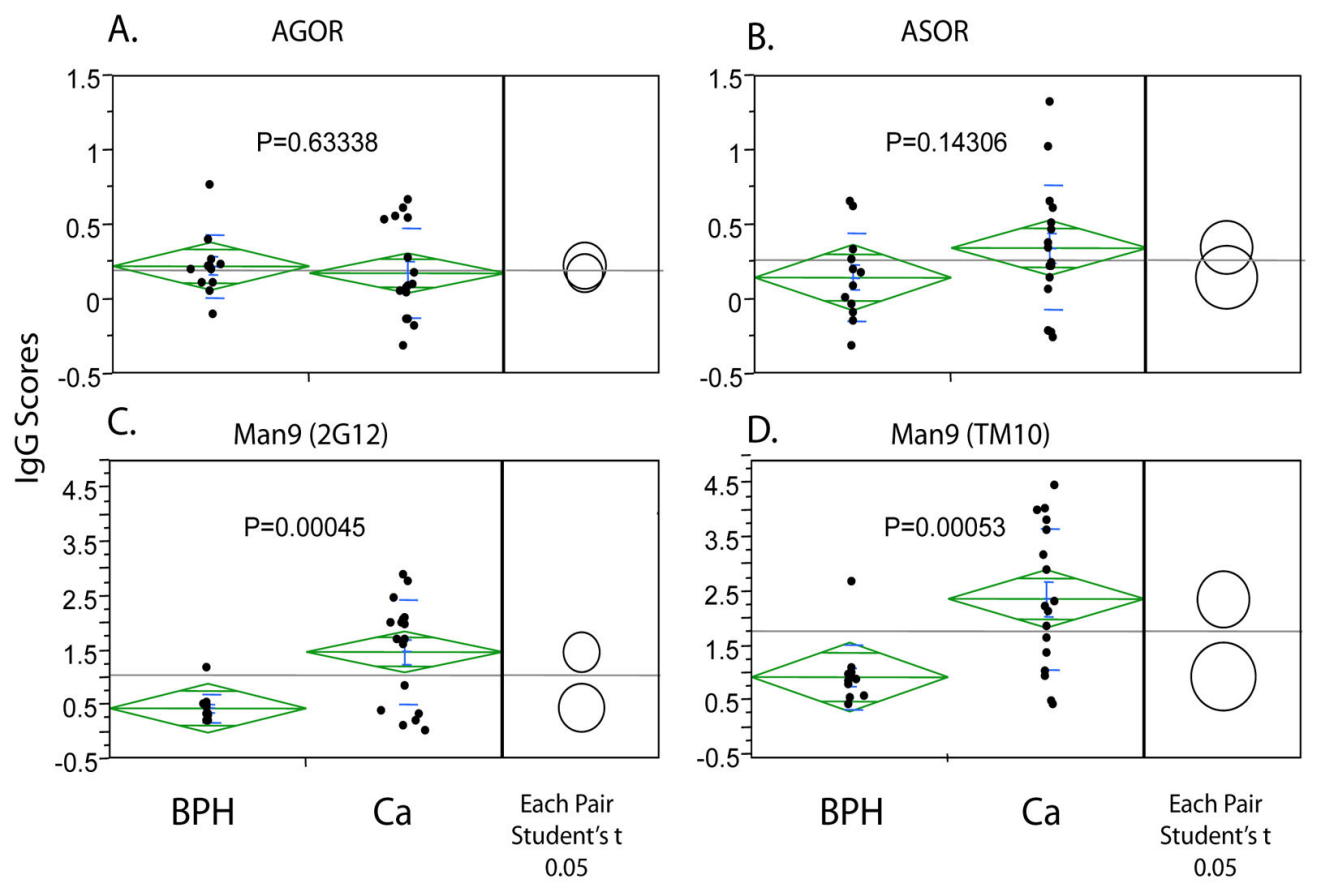
One-way analysis of anti-glycan autoantibodies between the PCa group (N=17) and the BPH group (N=12) Autoantibody reactivity with *N*-glycan cryptic antigen, AGOR (**A**), ASOR (**B**), and Man9 clusters (**C and D**) are generally present but are selectively enhanced to target the Man9-cluster epitopes in sera from prostate cancer patients (**C and D**).

The first step of this carbohydrate microarray analysis was to characterize the global antibody profiles of all subjects. These profiles were measured as the relative serum antibody reactivities (IgG scores) with a diverse panel of antigens spotted in the same carbohydrate microarray (Figure 2). Statistical analyses were followed to identify the glycan markers that detect significant levels of autoantibodies in human circulation (Figure 3) and further to those that capture PCa-associated autoantibody signatures (Figure 4).

In Figure 2, the antibody profiles are illustrated as overlay plots with IgG scores on the Y-axis and antigen probes aligned on the X-axis. Each colored symbol represents the IgG score of a given antibody specificity detected from a sera sample. The anti-self-antibody activities, including anti-carbohydrates and anti-lipids, are plotted to the left of a dashed line. Anti-non-self-antibodies, mainly anti-microbial polysaccharides, are plotted to the right of the line. Although the sera from the BPH and PCa subjects illustrate general similarity in their global antibody profiles, antibody reactivities targeting Man9 clusters spiked in the PCa sera (Figure 2A).

Figure 3 and 4 further examined statistically significant detection of autoantibodies by this microarray assay. In these figures, each dot represents the mean value of triplicate array detection of a sample. The mean of given group of subjects (represented by the green bar) and standard deviations (shown as a green diamond around the mean value) are shown to describe the size of the difference in relation to the distribution of values in each group. The comparison circles for Student’s t-tests appear to the right of the diamonds. The intersection of the circles illustrates the significance of the difference; the more the circles intersect, the less the significance of difference and vice versa.

Figure 3 addressed whether a given autoantigen detected antibodies in human sera. One-way analysis was performed to examine the significance of the differences of IgG scores among pairs of antigens. For examples, we examined pairs of OR-AGOR, OR-ASOR and AGOR-ASOR, respectively. OR, ASOR and AGOR are identical in their protein component but differ in the glyco-epitopes they express. AGOR and ASOR display Tri/m-Gn and Tri/m-II, respectively; OR do not surface-display either of the two glyco-epitopes. Thus, their pair-wise comparison is sufficient for revealing the carbohydrate-specific binding signals. As shown in Figure 3A and 3B, OR did not detect measurable antibodies. By contrast, its asialo form, ASOR (Tri/m-II) and agalato form, AGOR (Tri/m-Gn) captured highly significant amounts of IgG antibodies.

Figure 3C and 3D show one-way analysis among KLH, Man9 (2G12) and Man9 (TM10). These antigens share the KLH carrier but differ in carbohydrate units. Man9 (2G12) and Man9 (TM10) display Man9 in different cluster configurations. By comparing antibodies captured by these conjugates with those of the KLH control, it was determined that the two Man9 clusters detected highly significant amounts of IgG antibodies in both the PCa and BPH groups.

Figure 4 examined whether a detected serum autoantibody differed significantly between the PCa and BPH groups in order to identify potential “signatures” of PCa. For this purpose, autoantibodies detected in the PCa group were compared to those detected in the BPH group. The two Man9 clusters detected significantly increased levels of autoantibodies in the PCa group (Figure 4C and 4D). Man9 (TM10) appears to be more effective than Man9 (2G12) in capturing serum IgG. AGOR and ASOR did not detect significantly different levels of IgG between the PCa and BPH groups. However, a wide distribution of IgG^ASOR^ scores among samples from PCa patients was observed with a significant proportion of samples producing elevated levels of IgG^ASOR^ (Figure 4B).

## Discussion

In this study, we examined a panel of tumor-associated cryptic carbohydrate antigens for their potential immunogenic activities in humans. Our interest was to identify the carbohydrates that are able to trigger anti-carbohydrate antibody responses *in vivo*. Our assumption is that tumor carbohydrates with such immunological properties are suitable for development of tumor immunoassays and targeted immunotherapy.

The repertoire of human serum antibodies is broad and diverse [33]. How to standardize microarray datasets so that antibody profiles of different subjects can be quantitatively measured and meaningfully compared was a technical challenge to this study. We introduced the concept of Relative Antibody Reactivity (RAR) to standardize the global antibody profiles. In this way, the antibody profiles in comparison are independent of the serum antibody concentration, which is often subjected to the influences by a subject’s physiological or pathological status in having blood drawn in the clinic.

Figure 2 presents the RAR profiles captured for all tested sera samples, including those from both PCa patients (Figure 2A) and BPH subjects (Figure 2B). Visual inspection of this figure readily reveals that the global patterns of antigen-specific antibodies are impressively similar across subjects in both BPH and PCa groups. However, spikes of autoantibody activities emerged against the background of the conserved global antibody profiles. Interestingly, a number of spikes were identified in the same antigen ID# locations in the PCa- and BPH-plots. These are antigen ID #10, #12 and #26, which are Man9(2G12), Man9 (TM10) and cardiolipin, respectively.

Based on this observation, we infer that preexisting autoantibodies contribute to the enhanced antibody responses to tumor carbohydrates. Further investigation is required to determine whether autoantibodies produced by PCa patients, especially those with aggressive PCa, carry specific molecular signatures, such as selectively used germ-line genes or hyper-mutations in IgV regions. Practical approaches to address these issues include establishment of immobilized human B cell clones or determination of IgV region sequences by cloning and sequencing them from single antibody-positive B cells [34–38]. These approaches support recovery of the original pairs of V_H_ and V_L_ segments, which is essential for examining the clonal relationship among cells of different binding affinities with and without somatic mutations.

In the background of the RAR-standardized global antibody profiles, we further examined statistically meaningful detection of autoantibodies in human circulations. This effort is exploratory since it hits an immunological paradigm regarding human immune recognition of self-carbohydrate antigens. Figure 3A and 3B show one-way analysis of the IgG scores associated with OR, ASOR or AGOR. OR is an abundant human serum glycoprotein that is also known as α1-acid glycoprotein. As expected, this assay did not detect antibody to this fully glycosylated native human serum protein. By contrast, carbohydrate microarrays detected significant levels of anti-ASOR (IgG^ASOR^) and anti-AGOR (IgG^AGOR^) antibodies in sera from both BPH and PCa patients. Since OR, ASOR and AGOR have identical protein components, the differential antibody activities associated with them reflects glycan-binding specificities. As illustrated in Figure 1, corresponding cryptic moieties of OR were marked by other sugar moieties. Thus, IgG^ASOR^ captured by ASOR is predominantly Tri/m-II-specific; IgG^AGOR^ detected by AGOR reflects binding of Tri/m-Gn.

Detection of IgG^ASOR^ in human circulations is of particularly interesting since their targeting carbohydrates, Tri-/m-II, are characterized by having β(1,6)-branched type II chains. Hyper-expression of β(1,6)-branched type II chains, as determined by PHA-L staining, has been found in a number of human cancers [39]. Tissue-expression of this marker was also confirmed in the majority of PCa [16,17]. In viewing Figure 4B, we further noticed that a significant number of dots of IgG^ASOR^ in the PCa group are located above the upper 95% confidence limit line of the BPH group. This indicates that while the group means between PCa and BPH did not differ significantly (P=0.14306), a subset of PCa patients produced significantly increased levels of IgG^ASOR^ compared with the BPH group. The IgG^ASOR^ specificities were also found in SARS-CoV neutralization antibodies [21]. It would be highly interesting if SARS-CoV neutralizing antibodies bind to and effectively kill the Tri-/m-II-positive tumor cells.

Figure 3C and 3D show that the two Man9 clusters captured substantially increased levels of IgGs compared to those detected by their common KLH carrier molecule. [(Man9)4]n-KLH preserves well the HIV-1-specific Man9-glyco-epitopes recognized by 2G12. (Man9) n-KLH is poorly reactive with 2G12 but is highly reactive with the anti-tumor mAb TM10. Since IgG scores associated with Man9 (TM10) are significantly higher than the IgG scores of Man9 (2G12), IgG^Man9^ detected in human sera are likely dominated by TM10-like anti- Man9-cluster antibodies. Figure 4 illustrates further that both Man9 (TM10) and Man9 (2G12) detected significantly increased amounts of IgG antibodies in sera from the PCa group compared to sera from the BPH group. The Man9 (TM10) conjugate is the most effective one in capturing serum IgGs among all autoantigens examined in this microarray analysis. In the context of tumor cell-surface expression of the TM10-antigens [24] and intensive expression of oligomannoses in the higher Gleason grade cancers [16], IgG^Man9^ appears to be an attractive serum biomarker for further investigation.

In summary, we have found in this study that autoantibodies targeting the Man9- or Tri-/m-II-glyco-antigens are readily detectable in human sera and that these antibody reactivities were selectively increased in PCa subjects. An extended analysis of a larger cohort of PCa and BPH subjects is clearly required to determine the diagnostic and prognostic value of these autoantibodies. However, current study has sufficiently demonstrated that human immune systems actively recognize the Man9- and Tri-/m-II-expressing autoantigens and produce targeting antibodies. Since these carbohydrate moieties are generally “cryptic” but become highly expressed or surface-exposed in a number of tumors, it is worthwhile to further investigate their potential for the targeted immunotherapy against corresponding tumors. Much remains to be learned about the mechanisms of the host immune recognition and antibody responses to this class of cryptic glyco-antigens.

## Supplementary Material

Supplementary data

## Abbreviations

OR: Orosomucoid
ASOR: Asialo-Orosomucoid
AGOR: Agalacto-Orosomucoid
Tri/m-II: Tri-Antennary and Multivalent type II chains
Tri/m-Gn: Tri-Antennary and Multivalent GlcNAc cores
PHA-L: Phaseolus Vulgaris-L lectin
SNA: Sambucus Nigra I Agglutinin
GNA: Galanthus Nivalis Lectin
PCa: Prostate Cancer
BPH: Benign Prostatic Hyperplasia
