# Supplementary material for: N-glycan Cryptic Antigens as Active Immunological Targets in Prostate Cancer Patients
Source: J Proteomics Bioinform. Author manuscript; Available in PMC 2014 Oct 2. (PMC4183219; doi:10.4172/jpb.1000218)
Supplement: Supplementary data [file NIHMS569348-supplement-Supplementary_data.pdf]

Supplemantal Table 1 (to Figure 2)--Microarray dataset for seventeen prostate cancer and twelve BPH subjects						
**Antigens** (Reagent name, preparation code, and concentrations used for microarray printting)	Antigen ID#	Glycan array scores (IgG)				
		Prostate cancer (n=17)		BPH (n=12)		*t*- Test *
		Mean	SD	Mean	SD	(p values)
AGOR DW38 0.5mg/ml	1	0.179	0.303	0.225	0.209	0.63338
AGOR DW38 0.5mg/ml 1:5 in saline	2	-0.240	0.152	-0.177	0.135	0.25008
ASOR DW37 0.5mg/ml	3	0.348	0.416	0.149	0.295	0.14306
ASOR DW37 0.5mg/ml 1:5 in saline	4	-0.222	0.175	-0.186	0.125	0.52734
OR (1) DW749 0.5mg/ml	5	-0.281	0.158	-0.184	0.142	0.09732
OR (1) DW749 0.5mg/ml 1:5 in saline	6	-0.303	0.147	-0.234	0.123	0.17942
**[(Man9)4]n-KLH DW951 0.5mg/ml**	7	**1.478**	**0.963**	**0.441**	**0.264**	**0.00045**
**[(Man9)4]n-KLH DW951 0.5mg/ml 1:5 in saline**	8	**0.174**	**0.462**	**-0.127**	**0.119**	**0.01901**
**(Man9)n-KLH DW950 0.5mg/ml**	9	**2.367**	**1.304**	**0.927**	**0.597**	**0.00053**
**(Man9)n-KLH DW950 0.5mg/ml 1:5 in saline**	10	**0.561**	**0.701**	**0.047**	**0.290**	**0.01253**
**Man5-9-RB DW949 0.5mg/ml**	11	**0.148**	**0.298**	**-0.050**	**0.159**	**0.02902**
Man5-9-RB DW949 0.5mg/ml 1:5 in saline	12	-0.180	0.207	-0.172	0.096	0.88286
PtC L70905012 2mg/ml	13	-0.007	0.377	-0.025	0.157	0.86165
PtC L70905012 2mg/ml 1:5 in saline	14	-0.224	0.082	-0.179	0.081	0.15456
Sulfatide/PtC L70905-8 0.2mg/2mg/ml	15	0.067	0.285	-0.065	0.109	0.09704
Sulfatide/PtC L70905-8 0.2mg/2mg/ml 1:5 in saline	16	-0.193	0.174	-0.199	0.111	0.91021
Ceramide/PTC L70905-9 0.2mg/ml	17	-0.093	0.156	-0.096	0.096	0.93757
Ceramide/PTC L70905-9 0.2mg/ml 1:5 in saline	18	-0.227	0.098	-0.192	0.110	0.38436
Cerebrosides/PTC DW876 L70905-10 0.2mg/2mg/ml	19	-0.019	0.343	-0.101	0.087	0.35987
**Cerebrosides/PTC DW876 L70905-10 0.2mg/2mg/ml 1:5 in saline**	20	**-0.268**	**0.103**	**-0.182**	**0.101**	**0.03544**
Ganglioside/PtC DW866 L70905-4 0.2mg/2mg/ml	21	-0.009	0.253	-0.068	0.115	0.40170
Ganglioside/PtC DW866 L70905-4 0.2mg/2mg/ml 1:5 in Saline	22	-0.193	0.104	-0.159	0.111	0.41449
GM1/PtC L70905-3 0.02mg/2mg/ml	23	0.087	0.285	0.036	0.207	0.58106
GM1/PtC L70905-3 0.02mg/2mg/ml 1:5 in saline	24	-0.130	0.086	-0.092	0.127	0.38154
Cardiolipin/PTC L70905-2 0.4mg/2mg/ml	25	1.517	1.448	1.226	0.812	0.49720
Cardiolipin/PTC L70905-2 0.4mg/2mg/ml 1:5 in saline	26	0.191	0.193	0.113	0.212	0.32013
Cardiolipin/PTC L70905-1 0.1mg/2mg/ml	27	0.694	0.654	0.590	0.406	0.60242
Cardiolipin/PTC L70905-1 0.1mg/2mg/ml 1:5 in saline	28	0.117	0.181	0.021	0.075	0.06218
Glucocerebroside/PtC DW871 L70905-7 0.2mg/2mg/ml	29	-0.079	0.148	-0.122	0.089	0.34495
Glucocerebroside/PtC DW871 L70905-7 0.2mg/2mg/ml 1:5 in saline	30	-0.203	0.119	-0.197	0.105	0.87167
KLH-SH DW952 0.5mg/ml	31	0.273	0.385	0.067	0.175	0.06388
**KLH-SH DW952 0.5mg/ml 1:5 in saline**	32	**-0.251**	**0.144**	**-0.154**	**0.107**	**0.04831**
Phytosphingosine/PtC DW870 L70905 0.2mg/2mg/ml	33	-0.162	0.164	-0.165	0.102	0.95493
Phytosphingosine/PtC DW870 L70905 0.2mg/2mg/ml 1:5 in saline	34	-0.218	0.107	-0.242	0.146	0.63965
D-erythro-Sphingosine/ PtC DW867 L70905-5 0.2/2mg/ml	35	0.085	0.310	0.011	0.167	0.41267
D-erythro-Sphingosine/ PtC DW867 L70905-5 0.2/2mg/ml 1:5 in saline	36	-0.150	0.130	-0.166	0.115	0.73325
DMPS/PtC L70905-11 0.2mg/2mg/ml	37	-0.040	0.212	-0.035	0.142	0.94828
DMPS/PtC L70905-11 0.2mg/2mg/ml 1:5 in saline	38	-0.232	0.079	-0.181	0.108	0.17961
Yeast phosphomannan B2448 DW 41 0.5mg/ml	39	5.183	3.864	3.820	2.606	0.26686
Yeast phosphomannan B2448 DW 41 0.5mg/ml 1:5 in saline	40	1.381	1.075	1.001	1.012	0.34126
Dextran N279 DW49 0.5mg/ml	41	3.976	3.419	6.866	9.622	0.33697
Dextran N279 DW49 0.5mg/ml 1:5 in saline	42	2.594	2.302	4.497	6.248	0.33160
Dextran B1299S DW51 0.5mg/ml	43	3.861	4.786	4.438	6.773	0.80249
Dextran B1299S DW51 0.5mg/ml 1:5 in saline	44	0.979	1.672	1.535	2.820	0.54899
Dextran B1355S DW50 0.5mg/ml	45	1.701	1.501	2.996	1.925	0.06560
**Dextran B1355S DW50 0.5mg/ml 1:5 in saline**	46	**0.438**	**0.699**	**1.242**	**0.874**	**0.01553**
Levan DW42 0.5mg/ml	47	0.892	1.607	1.703	1.374	0.15690
**Levan DW42 0.5mg/ml 1:5 in saline**	48	**0.024**	**0.514**	**0.576**	**0.631**	**0.02092**
E. coli. LPS 5014 DW934 0.5mg/ml	49	0.365	2.313	0.951	1.805	0.45090
E. coli. LPS 5014 DW934 0.5mg/ml 1:5 in saline	50	-0.246	0.317	0.048	0.478	0.07881
E. coli. LPS 2630 DW933 0.5mg/ml	51	0.291	0.454	0.657	0.790	0.16729
E. coli. LPS 2630 DW933 0.5mg/ml 1:5 in saline	52	-0.094	0.278	0.142	0.379	0.08278
E. coli. K1 DW34 0.5mg/ml	53	1.564	1.338	1.192	1.305	0.46161
E. coli. K1 DW34 0.5mg/ml 1:5 in saline	54	-0.060	0.331	0.095	0.795	0.53541
E. coli. K100 DW36 0.5mg/ml	55	3.108	2.824	4.251	3.094	0.32087
E. coli. K100 DW36 0.5mg/ml 1:5 in saline	56	0.741	1.239	1.225	1.041	0.26494
E. coli. K92 DW92 0.5mg/ml	57	0.204	0.518	0.023	0.281	0.23883
E. coli. K92 DW92 0.5mg/ml 1:5 in saline	58	-0.280	0.156	-0.211	0.111	0.17709
S. dysenterine type I O-SP DW 765 0.5mg/ml	59	2.344	2.377	2.696	3.009	0.73871
S. dysenterine type I O-SP DW 765 0.5mg/ml 1:5 in saline	60	1.205	2.152	1.012	1.927	0.80189
S. typhi LPS7261 DW932 1:5 in saline	61	3.594	4.810	3.021	2.709	0.68697
S.Typhi LPS7261 DW932 0.5mg/ml	62	6.126	6.838	5.600	5.634	0.82255
Bacto-Agar DW801 0.5mg/ml	63	6.045	3.046	6.076	4.727	0.98460
Dye mix. (Cy3, Cy5, FITC-AV 1:100)	64	16.944	8.538	14.220	8.295	0.39797

* Results with significant difference (t-test, $p<0.05$) between prostate cancer group and BPH group were highlighted with **BOLD**.
